# Endoscopic ultrasound-guided reintervention for biliary metal stent kinking using a device delivery system

**DOI:** 10.1055/a-2155-4999

**Published:** 2023-08-31

**Authors:** Kei Yane, Masahiro Yoshida, Kota Hanada, Kotaro Morita, Hideyuki Ihara, Tetsuya Sumiyoshi, Hitoshi Kondo

**Affiliations:** Department of Gastroenterology, Tonan Hospital, Sapporo, Hokkaido, Japan

Kinking of the stent is one of the major causes of biliary metal stent dysfunction 1 . Although the preferred treatment is to remove the stent and deploy a new one, this is difficult to do in patients with duodenal obstruction due to tumor invasion. Herein, we report a case of successful endoscopic ultrasound (EUS)-guided reintervention for biliary metal stent kinking using a device delivery system.

A 60-year-old man was referred to our hospital for treatment of acute cholangitis and gastric outlet obstruction due to advanced pancreatic head cancer. Computed tomography showed stenosis of a previously deployed duodenal metal stent, bile duct dilation, and kinking of a biliary metal stent ( Fig. 1 ).

**Fig. 1:**
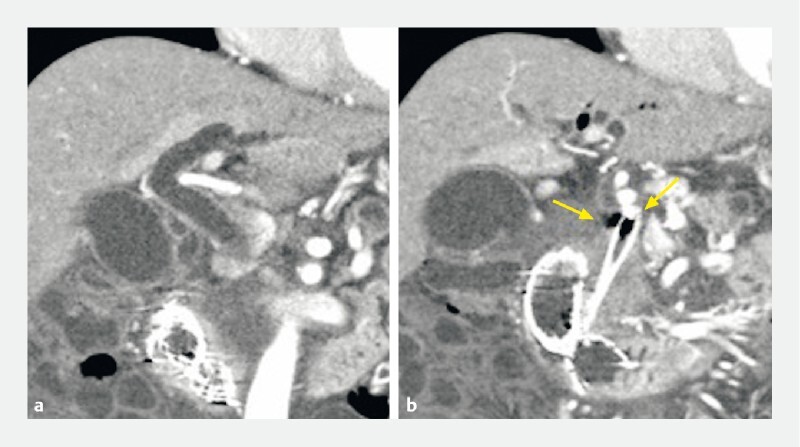
Computed tomography showed bile duct dilation and kinking of a biliary metal stent (arrows).

Following placement of an additional duodenal metal stent, biliary reintervention was performed. The dilated B3 was punctured with a 22-gauge fine-needle aspiration needle, then a 0.018-inch guidewire was advanced into the bile duct, and finally the fistula was dilated with a drill dilator. Cholangiography revealed that the bile outflow was blocked by the kinking of the biliary stent ( Fig. 2 ). A 0.025-inch guidewire was placed in the common bile duct, and the device delivery system (EndoSheather; Piolax Medical Devices, Kanagawa, Japan) was inserted. A biopsy forceps was passed through the delivery system lumen and the stent was grasped under fluoroscopic guidance. The proximal end of the stent moved towards the hilar side of the bile duct, the kinking was resolved, and the contrast material drained into the duodenum ( Fig. 3 ) ( Video 1 ). An additional metal stent was placed to prevent further kinking, and a plastic stent was placed at the fistula site ( Fig. 4 ). After the procedure, both cholangitis and gastric outlet obstruction improved rapidly.

**Fig. 2:**
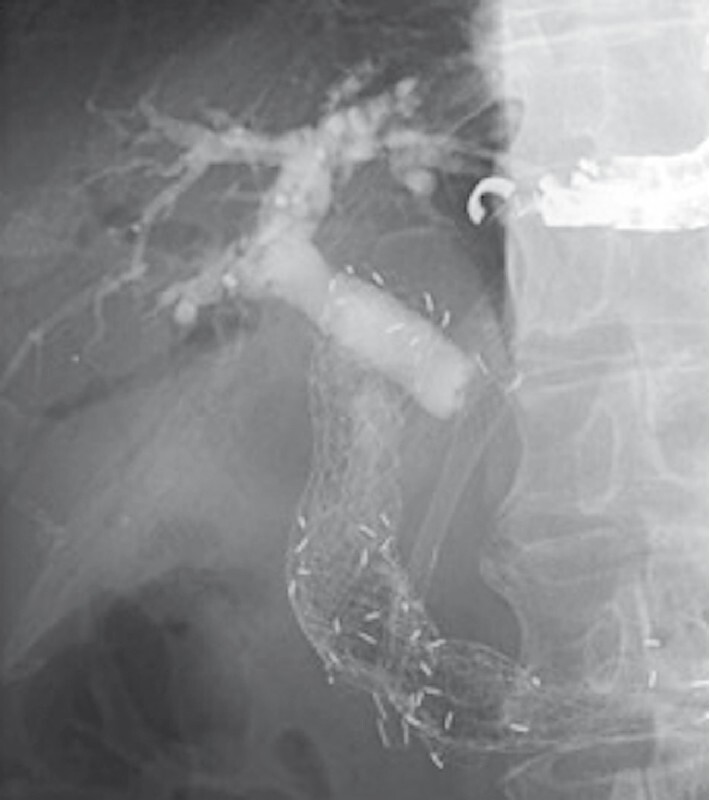
Cholangiogram revealed that the bile outflow was blocked by the kinking of the biliary stent.

**Fig. 3 a:**
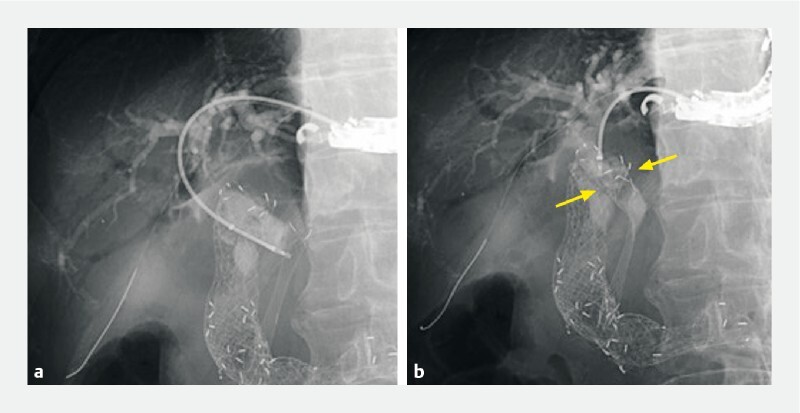
A biopsy forceps was passed through the delivery system lumen and the stent was grasped under fluoroscopic guidance. **b** The proximal end of the stent moved towards the hilar side of the bile duct (arrows).

**Video 1** A biopsy forceps was passed through the delivery system lumen and the stent was grasped. The proximal end of the stent moved towards the hilar side of the bile duct, and the kinking was resolved.


**Fig. 4:**
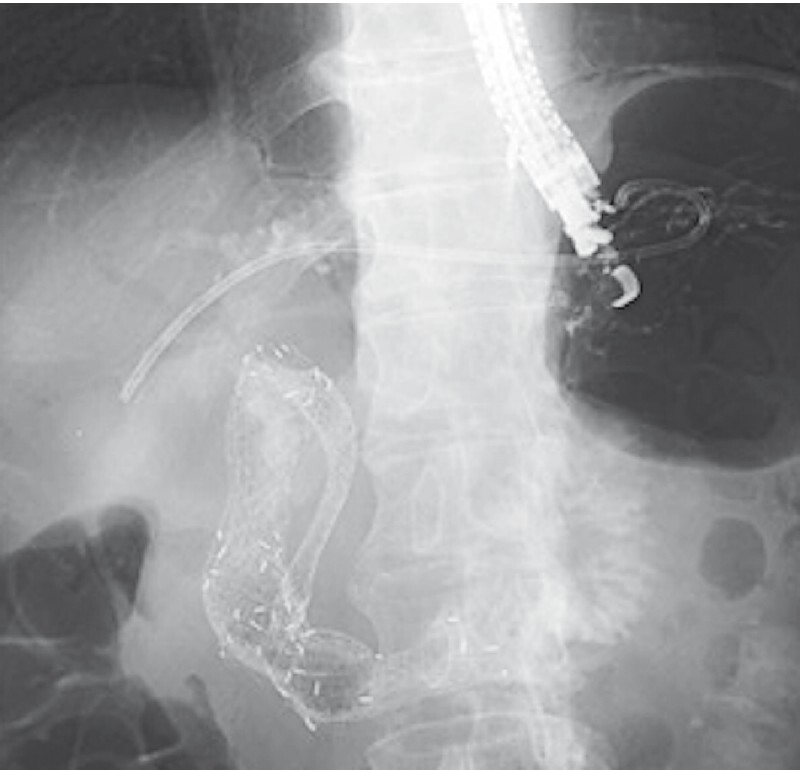
An additional metal stent was placed to prevent further kinking, and a plastic stent was placed at the fistula site.

Various devices, including biopsy forceps, can be inserted into the device delivery system 2 3 4 . This can also be performed without bile leakage from the hepaticogastrostomy site before the fistula matures 5 , and is a promising method for EUS-guided intervention.

Endoscopy_UCTN_Code_CPL_1AL_2AD

## Notes

Endoscopy E-Videos

https://eref.thieme.de/e-videos
*E-Videos* is an open access online section of the journal *Endoscopy* , reporting on interesting cases and new techniques in gastroenterological endoscopy. All papers include a high-quality video and are published with a Creative Commons CC-BY license. Endoscopy E-Videos qualify for HINARI discounts and waivers and eligibility is automatically checked during the submission process. We grant 100% waivers to articles whose corresponding authors are based in Group A countries and 50% waivers to those who are based in Group B countries as classified by Research4Life (see: https://www.research4life.org/access/eligibility/ ). This section has its own submission website at https://mc.manuscriptcentral.com/e-videos
